# Hybrid modeling techniques for 3D printed deep inferior epigastric perforator flap models

**DOI:** 10.1186/s41205-023-00181-z

**Published:** 2023-09-12

**Authors:** Nicholas M. Jacobson, Erik Carerra, Aaron Treat, Megan McDonnell, David Mathes, Christodoulous Kaoutzanis

**Affiliations:** 1 University of Colorado, Anschutz Medical Campus, School of Engineering, Computation, and Design - Inworks Innovation Initiative, Aurora, CO USA; 2 University of Colorado: Anschutz Medical Campus; School of Medicine; Plastic Surgery, University Hospital Colorado, Aurora, CO USA

**Keywords:** 3D Printing, Design, Medical Application, Software, Multi-Material Printing

## Abstract

**Background:**

Deep Inferior Epigastric Perforator Flap (DIEP) surgical procedures have benefited in recent years from the introduction of 3D printed models, yet new technologies are expanding design opportunities which promise to improve patient specific care. Numerous studies, utilizing 3D printed models for DIEP, have shown a reduction of surgical time and complications when used in addition to the review of standard CT imaging. A DIEP free flap procedure requires locating the inferior epigastric perforator vessels traversing and perforating the rectus abdominis muscle, perfusing the abdominal skin and fatty tissue. The goal of dissecting the inferior epigastric perforator vessels is complicated by the opacity of the fatty tissue and muscle. Previous attempts to 3D print patient specific models for DIEP free flap cases from CT imaging has shown a wide range of designs which only show variations of perforator arteries, fatty tissue, and the abdominis rectus muscle.

**Methods:**

To remedy this limitation, we have leveraged a voxel-based modeling environment to composite complex modeling elements and incorporate a ruled grid upon the muscle providing effortless ‘booleaning’ and measured guidance.

**Results:**

A limitation of digital surface-based modeling tools has led to existing models lacking the ability to composite critical anatomical features, such as differentiation of vessels through different tissues, coherently into one model, providing information more akin to the surgical challenge.

**Conclusion:**

With new technology, highly detailed multi-material 3D printed models are allowing more of the information from medical imaging to be expressed in 3D printed models. This additional data, coupled with advanced digital modeling tools harnessing both voxel- and mesh-based modeling environments, is allowing for an expanded library of modeling techniques which create a wealth of concepts surgeons can use to assemble a presurgical planning model tailored to their setting, equipment, and needs.

**Trial registration:**

COMIRB 21–3135, ClinicalTrials.gov ID: NCT05144620.

## Introduction

A cascade of technological advances in recent years has led to increased collaborations between surgeons and computational designers. This has produced a myriad of designs for 3D printed models to aid Deep Inferior Epigastric Perforator (DIEP) free flap surgery for breast reconstruction [1–4]. However, despite the widespread adoption of this procedure, variations between surgical sites, available tools, and expertise opens a gap for a variation of solutions. As a result, new technologies are necessary to grow the library of solutions surgeons can use to design custom 3D printed surgical planning tools. In doing so, 3D printing continues to deliver on its promise of providing personalized medicine.

This paper proposes a computational method for designing 3D printed DIEP models for surgical planning and decision making which has been developed by a team of computational designers and plastic surgeons at the University of Colorado Anschutz Medical Campus. Our proposed method builds upon previous attempts by adding a significant new technique, which can create multiple new concepts for biomedical modeling: multi-channel voxel modeling. The incorporation of our novel voxel-based modeling into the digital construction of the models allows inclusion of volumetric modifications that create precision measurements while maintaining morphological fidelity. Volumetric modeling methods for anatomy have been proposed before [5, 6], however our approach focuses on hybrid techniques for combining surface-based mesh modeling with voxel-based modeling. This method leverages voxel modellings’ native ability to composite, or ‘boolean’, complex geometries with minimal computational expense and employ kernel-based material specifications all while maintaining the ability to export models into the Stereolithography (STL) format for widespread 3D printing. The result of this hybrid process allows for complex geometric models to quickly and accurately blend, creating opportunities for what have traditionally been computationally expensive, multi-material anatomical structures, to be modeled and printed with ease and accuracy.

Previous 3D design approaches have demonstrated surgical benefits which include a reduction in operative time and increases in a surgeon’s confidence to perform the procedure [2]. An analysis of previous designs provides a glimpse into the different approaches, available equipment, and specific needs of each surgeon performing this routine and widespread procedure. These previous approaches all focus on visualizing and finding vasculature; however, the approaches have ranged from models which trace vessels across the skin to a focus solely on the muscle [1]. We hypothesize that apparent disconnection between various modeling solutions is a result of the inability to fully integrate all desired modeling elements due to computational modeling limitations. Our solution is beneficial in providing simple and computationally inexpensive methods to integrate the element of all previous attempts into one holistic model.

In this paper we discuss the issues and opportunities surrounding *image processing*. We then cover the methods for processes to *combine multimodal modeling engines*. Next, we show a technique for *voxel modeling* which includes the topics of *compositing, and* the construction of material channel-based *grids, which are used for precision measurements of the anatomy captured in the model.* Utilizing voxel modeling software, we provide an explanation of leveraging *soft body modeling* and the use of *export sequences* to achieve novel modeling abilities. *Printing and Post Processing* is discussed to complete the methods section, which demonstrates design opportunities within the fabrication process. Finally, we show the *results to the model and variational discoveries* and the *Use of 3D printed models in surgery* and *a Surgeon's perception of utilizing 3D models in surgery and* most importantly, how we approached *gaining trust.*

As demonstrated through this method, the incorporation of a voxel modeling environment with a traditional surface-based mesh modeling paradigm expands the design library for presurgical modeling. Voxel modeling environments demonstrate the ability for facile compositing of complex geometrical features and the addition of user defined material data incorporated into medical image derived anatomical 3D printed models. The use of this method is proven across a prospective 38 patient clinical trial approved by COMIRB #21–3135. This process expands the range of options a surgeon has in developing a model for their unique needs, individual workflows and available toolsets.

## Method and materials

Individual informed consent was obtained for all patients. All Digital Imaging and Communications in Medicine (DICOM) data was acquired from a new 32 channel Siemens Naeotom Computed Tomography (CT) scanner, providing the morphological base data for modeling. All data sets were acquired from living patients, during one imaging session and specific protocols were chosen for printing with the guidance of a Board Certified Radiologist.

### Combining multimodal modeling engines

**Fig. 1:**
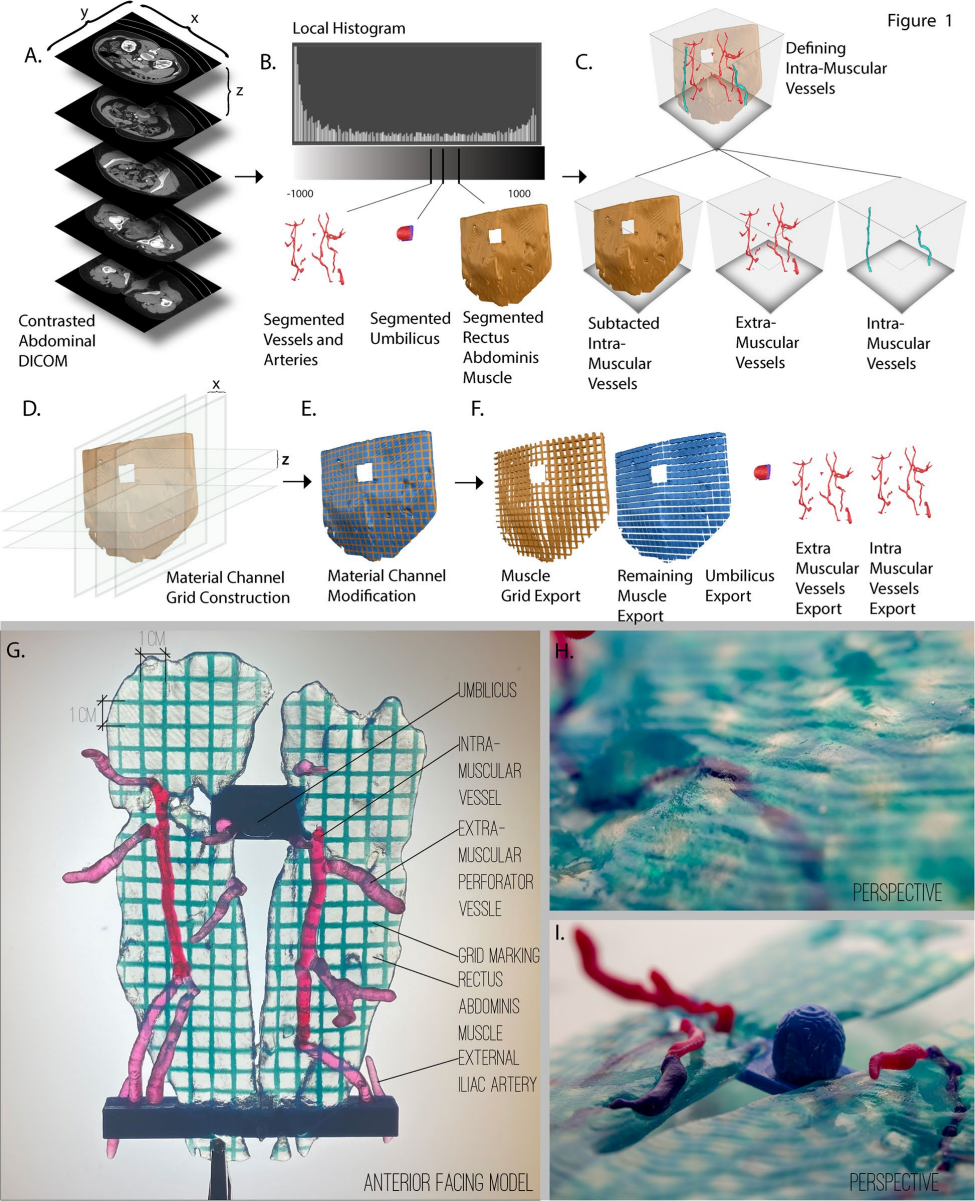
General workflow for the conversion and compositing of medical data through hybrid modeling techniques to generate 3D-printed tangible objects. **A** A single CT DICOM image stack is processed, and image-derived intensity values are calculated. **B** User-defined threshold is created at specific intensity values to segment specific anatomy. Segmentations are imported into a voxel-based modeling software where composting functions define the intra and extra-muscular sections of vessels. The resulting vessels are subtracted from the muscle creating voids for future 3D printing operations. **C** A voxel color channel modeling layer is applied above the muscle and composited down upon the muscle. User defined grids are modeled in the vertical and horizontal plane at even increments to define a regular grid. **D** The result of the color channel grid defined a differentiation in color only, leaving the morphology intact, which is later converted to an isosurface for exporting to a mesh. Finally, the grid, remaining muscle, intra- and extra-muscular vessels, and umbilicus are exported separately as mesh files for 3D Printing (**E**). **F** A singular model representation delineating the component anatomical features of the model. Detailed perspective highlights demonstrating the visualization of vessels variations (**G**) (**H**)

Our method utilizes a *hybrid digital modeling method which incorporates voxel-based modeling technology* with *surface mesh based modeling, the* most commonly used paradigm for digital modeling and the creation of objects for 3D printing [7, 8]. This method concludes with the creation of a multipart model which exports to the Standard Tessellation Language (STL) file format for polyjet based multi material 3D printing [2]. An overview of our first method is shown in Fig. 1. The development of this process was completed in collaboration with the attending surgeons performing DIEP free flap procedures at the University of Colorado Hospital on the Anschutz Medical Campus. Through an iterative process involving reviews of the digital workflow, the resulting models, and surgical observations, we arrived at the method described below.

### Image processing

CT DICOM files were first processed with the open-source 3D Slicer software [9, 10]. In this multi-step process, a level set thresholding technique was first employed to construct a segmented volumetric model of the desired anatomy per Jacobson et al [11]. Each anatomical feature was segmented into separate layers based on the corresponding Hounsfield range. Multiple discrete segmentations were edited using smoothing and freeform soft body modeling to further isolate individual models which included the rectus abdominis muscle, inferior epigastric perforator vessels (both deep and superficial when detected), external iliac artery, and umbilicus. The resulting segmentations were individually exported to STL using the OOTB export module in Slicer V10.4.2. This version of the 3D slicer processes the bounding mesh files utilizing the Flying Edges algorithm implemented by the VTK library [12]. In this process each voxel is converted to a volume bounded by triangulated facets, therefore the number and size of the facets are dictated by the voxel size of the segmentation label map representation of the geometry.

### *Voxel Modeling:* Muscle > Vessels > Umbilicus

The individually segmented STL models are then imported into Monolith v.0.3, a voxel-based modeling environment with a layer compositing system [13]. In the layer compositing system, where every layer generates voxel values that are subsequently combined with the corresponding voxel values below, a user-selected model is imported first to serve as a base layer and additional STL models are loaded into separate layers in a hierarchical structure. Using the layer compositing options interface, which roughly corresponds to mesh based boolean operations, the ‘Subtract’ operation is selected for top layer models to create voids in the base model. For our model, we specified the rectus abdominis muscle as the base layer, vessels as the second layer, and the umbilicus as the top layer.

### Voxel compositing

In the next set of operations, we utilized the layer compositing functions to specify material assignments. If the vessels are intramuscular and causing a subtraction in the rectus abdominis muscle, determined in the step above, a color channel based additive arithmetic composting operation is performed to modify that portion of the corresponding voxels associated to the intra-muscular vessels by color only, leaving the morphology unaltered. The resulting material differentiation specifying intra and extra-muscular vessels are exported as STL files by creating closed isosurface meshes at the midpoint of the material gradient between the rectus abdominis muscle and the intramuscular vessels.

### Voxel based grids

The penultimate modeling step integrates a user defined grid via material channel-based modeling additions. The purpose of the grid is to provide a precision measurements on the rectus abdominis muscle, which can provide coordinate locations of the perforating vessels. To achieve a grid across the rectus abdominis muscle, a color channel layer is added and composited to operate directly above the layer containing the rectus abdominis muscle and below the vessels. In this method a geometry-based layer creates a voxel field, through the material channel, controlled by a geometric primitive plane. Each plane generates a numerical halo that fades with distance. The strength and falloff of this halo will determine the blending behavior of the generated field. In this layer, a user-defined array of planes are created and spaced out both horizontally and vertically at a 1 cm increments, centered upon the umbilicus. The strength of the planes is set to 100% and the fall off distance is set to 1 mm, defining a consistent 2 mm wide plane with a 1 cm grid spacing. This layer is composed with an addition-based operation acting upon the rectus abdominis muscle base layer, superseding the base layer voxels with the intersecting grid voxel data.

### Soft body modeling and export sequences

Finally, free form soft body modeling is used to manually create support structures that are placed to support fragile vessels, thin areas of muscle, and to support the umbilicus. The resulting composited model is exported to STL. Each layer is exported individually at the mesh resolution of the voxel inherited from the source images. The two-color channel modifications, the grid and the intra-muscular vessels, are exported as closed meshes along the material mixing color channel threshold. In this algorithm, all pixels whose intensity level lies above the threshold value are quantized to material 1, all others are assigned material 2 and isosurfaces are created from the material ratio channel and trimmed by the shape channel.

### Printing and post processing

The amalgamation of STL models is loaded into a slicing environment for polyjet printing. The muscle file is set to a clear material and the accompanying grid is set to an opaque material. The intra and extra-muscular vessel segments are specified to be contrasting colors. The umbilicus and structural struts are set as another separate unique opaque color. The surface finish is set to high gloss and the print orientation is configured to reduce support material from the superior face of the rectus abdominis muscle. The resulting models created in Method 1 are fabricated using a commercially available Connex250 multi-material 3D printer (Stratasys, Rehovot, Israel). Depending on the desired color range of the printed objects, high-modulus, Vero series, photopolymers of specific colors, and transparencies were employed as the constituent materials. Post processing of support material is removed from the back side, however support material between the abdominis rectus muscle and the extra-muscular perforator vessels is not removed.

## Results

### Hybrid modeling modalities

**Fig. 2:**
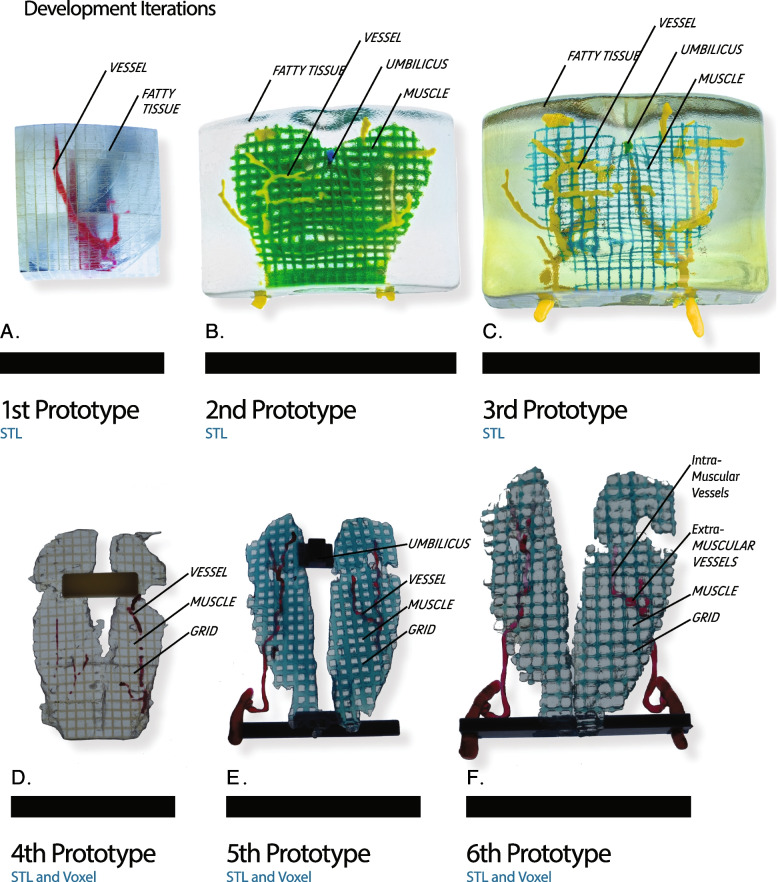
Numerous iterations of the model design were made with the surgical team. **A** The first iteration incorporated only the fatty tissue and perforator vessels. A grid was represented through a boolean subtraction leaving a perforated void throughout the model, volumetrically. **B** The second iteration incorporated the muscle with a 1 cm grid encased in a clear representation of the fatty tissue. **C** The third iteration focused on making the muscle grid thinner and translucent which allowed for a better visualization of the intramuscular vessels. **D** The fourth iteration eliminated the fatty tissue which was deemed unimportant and proved to hinder the 3D understanding of the extra-muscular vessels. **E** The fifth iteration focused on legibility of the grid which was rendered in a contrasting color to the vessels and incorporated the umbilicus as the origin of the grid. **F** The final iteration focused on a refinement of the fall off and strength of the voxel-based grid while incorporating a color-based differentiation between intra- and extra-muscular vessel locations

An overview of our resulting modelling development process is shown in Fig. 2. Utilizing the method above 38 DIEP flap models were fabricated for prospective surgical cases involving human subjects. The resulting models were reviewed prior to surgery under an IRB approved study per COMIRB 21-3135. All models were reviewed by a Board Certified Radiology prior to surgery.

### Imaging and modeling

**Fig. 3:**
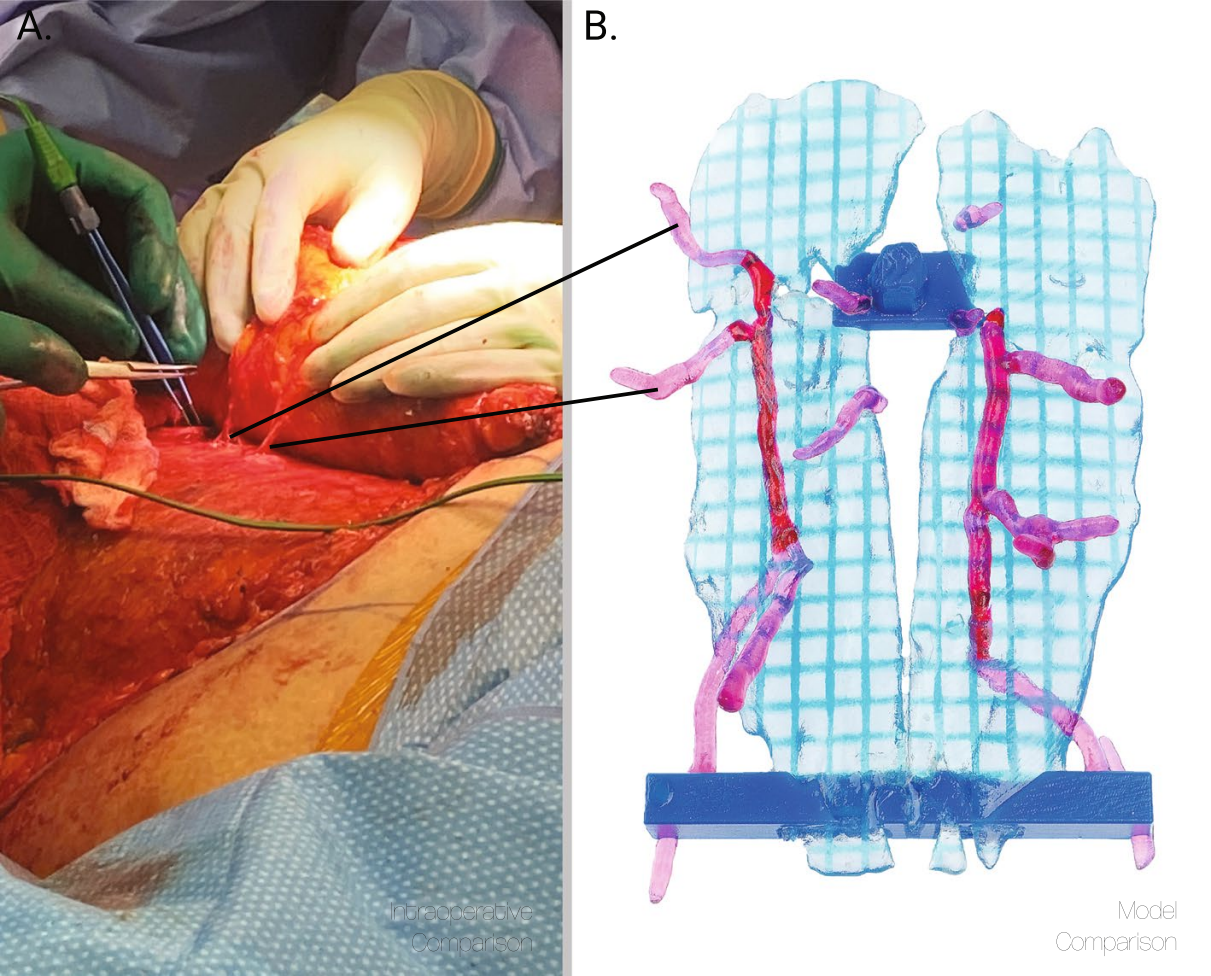
An intraoperative comparison of actual patients’ anatomy (**A**) with a 3D printed model replica (**B**). This is used to demonstrate and validate the accuracy of our process. All interoperative comparisons were taken qualitatively because due to the nature of the fatty tissues, the exact location is dynamic, and the approximate location is sufficient for this application

First, a contrasted 1 mm slice thickness, isotropic 512 × 512 pixel per inch resolution axial Maximum Intensity Projection (MIP), with isovist contrast, abdominal CT DICOM is visualized in 3D Slicer and used first to identify vessel locations for reference. Next, axial arterial phase 90 s submillimeter images were used to segment the muscle, vessels, and umbilicus. The segmentation of the rectus abdominis muscle was created by a level set thresholding a hounsfield range of between 180-230hu. Then a separate segmentation is created of the inferior epigastric perforator vessels, from the junction of the external iliac artery to the terminus of the vein and thresholded to a hounsfield range of 900–1100, dependent on contrast saturation along the length of the vessels. In areas where a vessel appears discontinuous, due to gaps with imaging slices, the MIP was consulted and vessels were manually modeled using a freeform digital modeling brush. To acquire this range, an intensity range sample is taken from the descending aorta, providing an average hounsfield number, which contains the same contrast saturations and provides a larger consistent sampling space. Manual gap filling and erasing adjustments are made to the rectus abdominis muscle and inferior epigastric perforators segmentations to ensure all elements are represented coherently. Finally, the umbilicus is manually segmented through the rectus abdominis muscle and infilled with a freeform modeling brush. The resulting model was compared intraoperatively as shown in Fig. 3.

### Variations

**Fig. 4:**
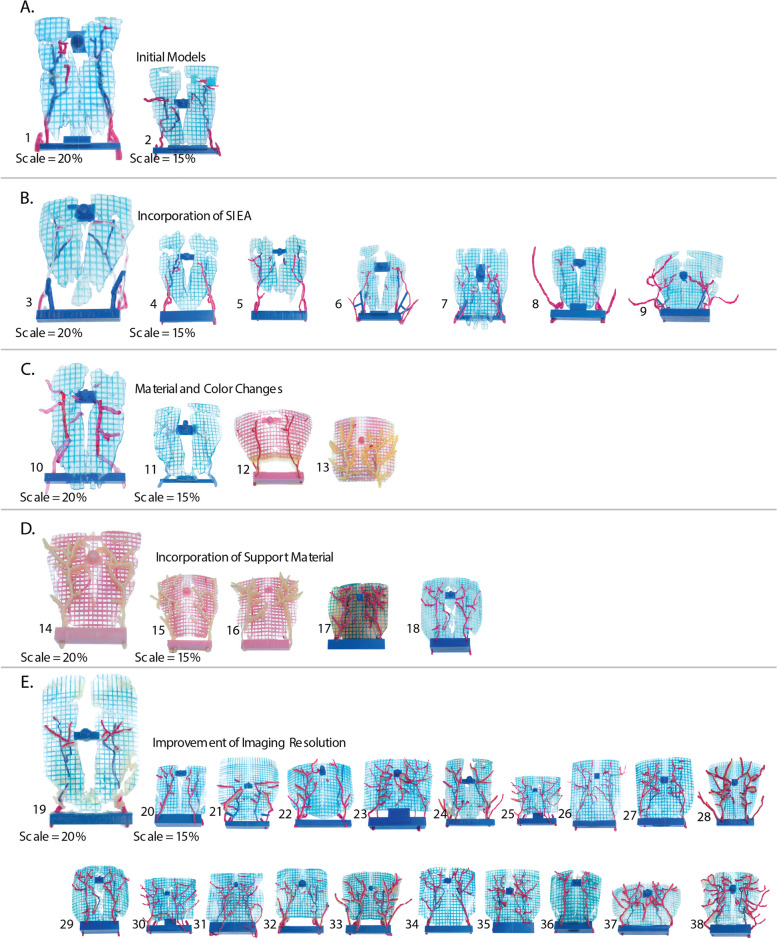
The evolution of models in the study separated by design developments and findings incorporated throughout the process (**A**), The initial models and final models varied slightly. **B** The first addition of incorporating SIEA’s was called for by the surgical team during a procedure and was incorporated in all models going forward. This demonstrates the ability for surgeons to directly translate difficulties into rapid solutions through in the moment conversations with the design team. **C**. A COVID-19 related shortage of material forced the design team to investigate different material combinations which ultimately proved more challenging to quickly comprehend. **D** Support material was left unremoved between the extra-muscular vessels and muscle, which provided a visual connection for the surgeon to better track extra-muscular vessel locations in the fatty tissue. **E** A coordinated series of conversations with the radiological team and surgical team lead to an increased scan resolution which provided a more accurate visualization of all perforator vessel present in the patient

The process consistently produced repeatable results with slight variations as seen in Fig. 4. Differences in muscle location and morphology, specifically when patients had prior surgeries which have disrupted the rectus abdominis muscle, such as a hysterectomy or an appendectomy, caused variation in the thresholding process, requiring the addition of manual edits with a freeform modeling brush. Superficial Inferior Epigastric Arteries (SIEA) were observed and captured in the process. SIEA dominant flaps are an alternative approach to flap harvesting which is uncommon but must be accounted for in the modeling process for surgical planning [14]. In these cases, additional care was given to ensure supports were placed and print orientation minimized support material to ensure the integrity of the delicate structures.

### Printing variations

The rectus abdominis muscle was 3D printed in a clear material, which allows for the ability to see the intra-muscular course of vessels and arteries. The grid was printed in an 80% mixture of cyan and 20% clear, which provided legible material differentiation without obstructing the ability to see intramuscular vessels. The vessels were printed in a mixture of 100% magenta for the extra-muscular section and an equal mixture of equal parts magenta and cyan, resulting in a purple coloration, for intra-muscular sections.

### Post processing

All models were printed with a glossy finish and post processed manually in a water blast cabinet. The first 5 models were sprayed with a high gloss clear coat finish using an airbrush. These models produced results that proved to reflect the glare of surgical lights, causing optical distortions. The remaining models were. The untreated models showed to reduce the levels of glare which are associated with operating room lighting, which provides a clearer view of the intramuscular vessel locations.

## Discussion

### Surgery

The creation of the method and models is the result of an iterative process between the surgical and design team similar to Zablah et Al [15]. Numerous designs were explored over the course of months, which involved a co-design process driven by our technological capabilities. Our process began with designs presented to the surgical team by engineers which facilitated the start of a conversation regarding the needs of this specific team. Based on the conversations with the surgical team, the design team worked to create numerous models which attempted to address the surgical needs. The design team took care to propose additional concepts that stemmed from a synthesis of those conversations, which sought to demonstrate new techniques resulting in new concepts. In subsequent meetings this process facilitated deep conversations about the intricacies of procedures providing the design team with more insight into the procedure. These conversations, surgical observations, and iterative process led to a honing of a design which synthesized the design team's technology and capabilities with the needs of the surgical team.

### Perception and trust

The use of models by the clinical team, despite being heavily involved in the design process, required mental adjustments. The first 5 models elicited skepticism from the clinical team as the accuracy of the perforator locations. Once the team validated the locations against the images and the model was found to be accurate, the team routinely reported surprise by the visual location of anatomical features after reviewing imaging. This outcome reflects the changing analysis methods a clinician undergoes when transitioning from 2D computer graphics to a tangible 3D spatial representation of the same data [4].

### Modeling

This hybrid method of modeling allowed for an expanded language by which surgeons could design a model to address a greater swath of individualized needs. Our hybrid process creates solutions to the roadblocks related to boolean operations associated with STL printings. This can be done with a surface-based mesh approach using a boolean operation, however, this process can be computationally prohibitive and explains why few, if any, anatomical models have been 3D printed involving vessel booleaning. Utilizing mesh-based modeling, the resolution of polygons is proportional to the number of bits used to represent polygons but the resolution required to boolean numerous vessels, multiplies the number of bits required due to calculating intersections between multiple meshes. Therefore, this process is computationally prohibitive and would require 1 terabytes of processing and 32 cores to handle the patching of memory, whereas our method can produce accurate results in under 15 s utilizing 100 gigabytes of processing and 16 cores. In total, the process from image to print file is generally 90 min, dependent on the complexity of the anatomy and the skill of the technician. Furthermore, the marching cubes and flying edge algorithm driving booleaning creates faceted intersections disturbing the morphological features and introducing error and volume loss as described by Jacobson et Al [16]. Voxel modeling provides a computationally inexpensive alternative to ‘booleaning’ through composting functions calling a regular grid of voxels which additionally ensures a watertight split between exported STL files [17].

### Limitations

**Fig. 5:**
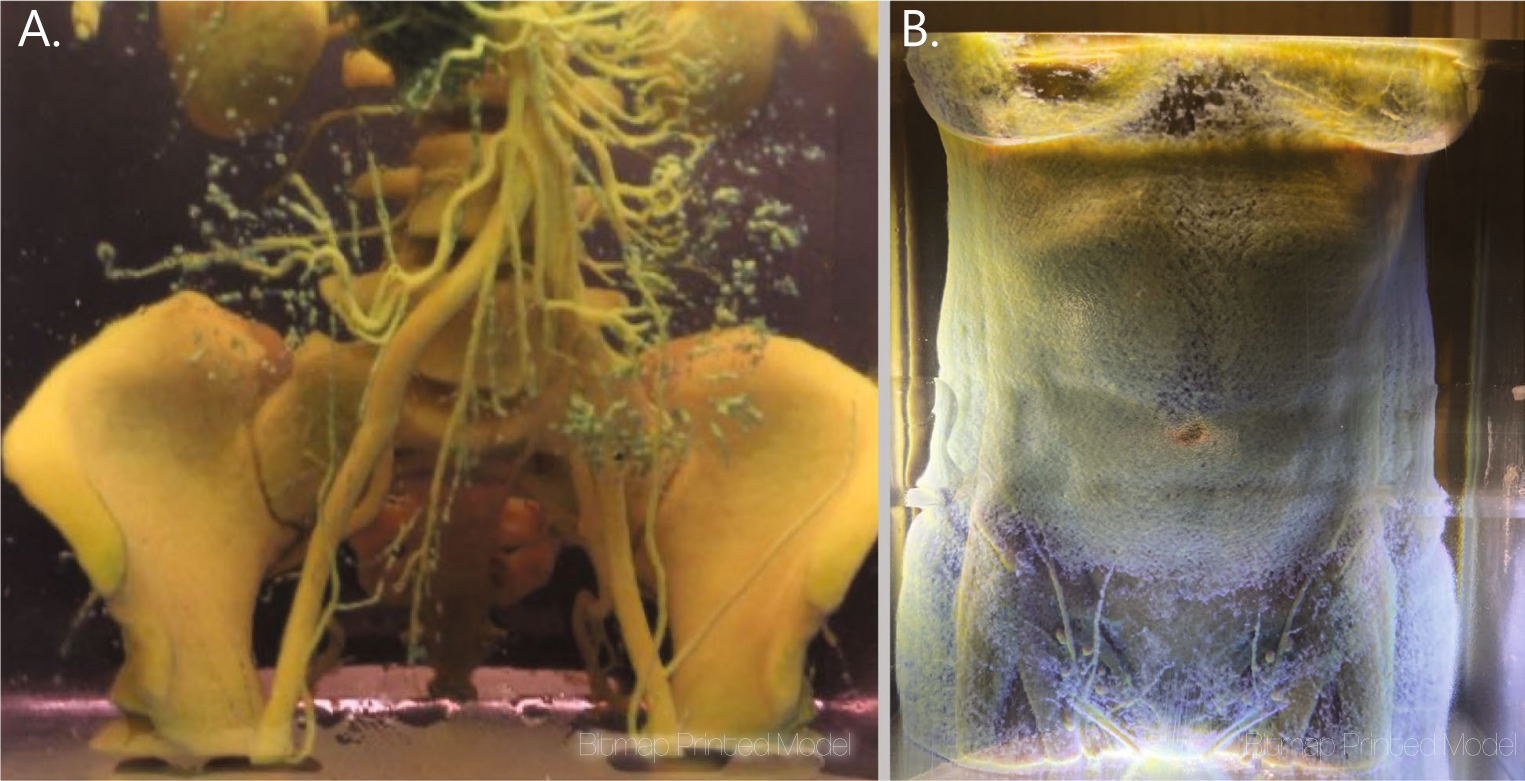
A bitmap printing exercise used in early conversations to assist with design concepts. **A** A bitmap printed abdomen filtered to highlight the bone and contrasted vessels. A lower intensity value filter applied to the same data to represent the vessels and muscles with a thin translucent layer of skin **B**

As with all medical 3D printing, the process is dependent upon the quality of the input data, in our case the abdominal DICOM images. To fix problems related to gaps in imaging or the result of artifacts and anomalies such as scar tissue, as mentioned above, liberties can be taken based on educated assumptions to edit the digital models, however these assumptions are limited. For example, we routinely discovered perforator vessels during surgery which did not show up on the scans. Sadly, there is little, if anything, that can be done on the modeling side to correct this data gap. A study of the use of MRA scans has shown promise for capturing a higher percentage of vessels than the standard CTA [18]. The use of MRA scans in our process would constitute a trivial change and holds promise for improving the results of this process. Furthermore, vessel diameter is a critical element surgeons use when developing a preoperative plan. Since the segmentation process utilizes the marching cubes algorithm based on inclusion criteria for the resulting STL model, it is not possible to accurately capture the size of such small features. Bitmap printing of perforator vessels was explored in nascent stages during this research and showed promise for directly translating vessels from images bypassing the mesh-based segmentation process (Fig. 5). Further studies are currently underway to quantify the accuracy between the diameter sizing of vessels represented in bitmap printing to in situ vessels measured during surgery.

## Conclusion

The methods described herein show the utility of a hybrid modeling approach merging surface mesh-based modeling with voxel modeling. The ability to do so has expanded the range of features and control a surgeon has in designing a model to their individual needs. Putting the power of design in the hands of the front-line staff utilizing these tools is critical to increasing confidence in their surgical approach and improving patient outcomes. The results of this increased confidence may reduce operating time and complications associated with a procedure. Computationally, this hybrid modeling approach overcomes issues related to surface mesh-based modeling in relation to boolean operations and effortlessly allows for the merging of multiple modeling elements regardless of topological complexity. The result of this technique creates the opportunity for the integration and interweaving of more complex model elements. Finally, we show the ability to simply apply kernel-based modeling operations across select elements allowing for data to be integrated within a model element without topological alterations. The use of augmented reality would be a suitable substitute for 3D printed models in this application. The ability to overlay precision measurement elements within a printed object allows for the model's utility in more procedures which require precision in biologically opaque areas such as the brain, anterolateral thigh flaps for the creation of phalloplasty’s, and genioplasties for facial reconstruction surgery for gender affirmation.

## Data Availability

All data will be provided by the author upon request. Nicholas.Jacobson@Cuanschutz.edu.
